# Continuums of Change in a Competence-Building Initiative Addressing End-of-Life Communication in Swedish Elder Care

**DOI:** 10.1177/10497323211012986

**Published:** 2021-05-13

**Authors:** Therese Johansson, Carol Tishelman, Joachim Cohen, Lars E. Eriksson, Ida Goliath

**Affiliations:** 1 Karolinska Institutet, Stockholm, Sweden; 2 Stockholm Healthcare Services, Region Stockholm, Stockholm, Sweden; 3 Vrije Universiteit Brussel & Ghent University, Brussels, Belgium; 4 City University of London, London, United Kingdom; 5 Karolinska University Hospital, Stockholm, Sweden; 6 Stockholm Gerontology Research Center, Stockholm, Sweden

**Keywords:** death literacy, participatory action research, advance care planning, staff education and training, nursing homes, qualitative research, longitudinal qualitative analysis, Sweden

## Abstract

Conversations about values for the end-of-life (EoL) between residents, relatives, and staff may allow EoL preparation and enable value-concordant care, but remain rare in residential care home (RCH) practice. In this article, longitudinal qualitative analysis was used to explore changes in staff discussions about EoL conversations throughout workshop series based on reflection and knowledge exchange to promote EoL communication in RCHs. We identified three overall continuums of change: EoL conversations became perceived as more feasible and valuable; conceptualizations of quality EoL care shifted from being generalizable to acknowledging individual variation; and staff’s role in facilitating EoL communication as a prerequisite for care decision-making was emphasized. Two mechanisms influenced changes: cognitively and emotionally approaching one’s own mortality and shifting perspectives of EoL care. This study adds nuance and details about changes in staff reasoning, and the mechanisms that underlie them, which are important aspects to consider in future EoL competence-building initiatives.

## Introduction

Dying today often is an expected and gradual process (Walter, 2017). As is increasingly the case in many Western countries, residential care homes (RCHs) are common sites for end-of-life (EoL) care provision in Sweden, with >36% of all deaths occurring there (Håkanson et al., 2015; Swedish Register of Palliative Care, 2020). Although many older adults value the possibility to discuss thoughts and feelings about the EoL, such conversations between staff and residents are rare (Baranska et al., 2020; Fleming et al., 2016). Similarly, a “discourse of silence” surrounding the EoL has been noted in Swedish elder care (Österlind et al., 2011). Avoiding EoL issues may hinder preparation for dying among residents and their relatives (Omori et al., 2019; Österlind et al., 2016). In light of the impact of the COVID-19 pandemic on mortality in elder care, EoL communication deficits have become further highlighted. In contrast, early opportunities for residents to reflect on, identify, and discuss their EoL values and preferences with care staff and/or relatives are important prerequisites for value-concordant future care (Howard et al., 2015). EoL conversations have been shown to reduce the risk of unwanted care procedures and increase relatives’ involvement in, preparedness for, and satisfaction with, EoL care (Barken & Lowndes, 2018; Supiano et al., 2019).

Internationally, discussions to identify and document preferences for EoL care are known as advance care planning (ACP) (Jimenez et al., 2018). However, ACP often focuses on creating legally binding documents, that is, advance directives, or appointing legal proxies, none of which is legally admissible in Sweden at present. Consequently, this article addresses EoL conversations only and not documentation of care preferences. While ACP is not implemented in Swedish care, the national guidelines for palliative care recommend physician-led discussions about EoL care options with patients and their relatives (National Board of Health and Welfare, 2016). However, these discussions, which often focus on ending medical treatment and provision of comfort care, usually occur at a late stage, when death is imminent (Udo et al., 2017). A recent report from the Swedish Palliative Care Register, containing information about care provided the final week of life, shows that in 2019, physician-led EoL conversations were offered in connection to 77.6% of registered deaths in RCHs, though it remains unclear how many of these were actually performed and whether discussions were held with residents themselves or their relatives (Swedish Register of Palliative Care, 2020).

Whereas death is perceived as a natural part of life in RCHs, elder care staff in Sweden have been found to be reluctant to engage in EoL conversations, possibly due to experienced social taboos (Alftberg et al., 2018; Holmberg et al., 2019). Staff’s ability to respond to and speak about residents’ thoughts about dying and death has, thus, been identified as a growing challenge for elder care (SOU, 2017). Similarly, international research on staff attitudes to EoL conversations illustrates challenges; while EoL communication with care recipients and relatives is considered an important aspect of care, staff often find it difficult to address the EoL and may avoid the topic altogether (Almack et al., 2012; Broom et al., 2014). In the RCH setting, there is a particular need for EoL communication training (Chung et al., 2016), as assistant nurses (ANs), who are primary caregivers to dying residents, often lack training in, and confidence for, discussing EoL matters (Frey et al., 2019). Reflecting on dying and death, including one’s own mortality, has been proposed one means of preparing RCH staff to support residents at the EoL, for example, by enhancing self-awareness of own emotions in relation to death (Österlind et al., 2011). Reflection is a central tenet in care staff education and skill development, often conceptualized as critical analysis of knowledge, experiences, or emotion, to achieve deeper meaning and understanding (Mann et al., 2009). Similarly, knowledge exchange, a multidirectional and dynamic process, which incorporates sharing and learning from both explicit and tacit knowledge, is another means for professional development and informing change in practice (Ward et al., 2012). Thus, reflection and knowledge exchange can both be considered mechanisms that trigger social learning and enable integration and development of research-based and practice-based knowledge (Nilsen et al., 2012).

As part of a collaborative effort to improve EoL care provision with Stockholm City Elder Care Bureau, the municipal agency responsible for all elder care in Stockholm, we used the existing evidence on reflection and knowledge exchange to organize and conduct series of workshops with elder care staff to build EoL-related competence. The aim of this article is to investigate changes in workshop series designed to promote EoL communication competence by exploring staff reasoning and discussions about EoL conversations in elder care.

## Method

### Study Design

This longitudinal, explorative qualitative analysis is based on data from a larger participatory action research (PAR) project exploring prerequisites for proactive EoL conversations in elder care, carried out within the national DöBra* research program (Lindqvist & Tishelman, 2016). The DöBra program uses various participatory approaches with the goal of enabling people to be better prepared for the EoL. PAR studies build on an iterative process conducted in collaboration with stakeholders, which involves cycles of planning, action, reflection, and evaluation, to understand and improve practice in specific contexts (Baum et al., 2006). In line with PAR principles, local management in participating services collaborated through shared planning meetings at each site to determine workshop content and discuss local needs. The study was approved by the Swedish Ethics Review Authority (reference number 2017/488-31/4).

### Participants and Study Setting

We used purposive sampling in a two-phased recruitment process, first recruiting services and then individual staff members from them. In the first phase, two elder care district managers at the Stockholm City Elder Care Bureau agreed to facilitate contact with potential research sites. We strove for an inclusive PAR process to explore EoL communication in a variety of contexts and from various perspectives. Subsequently, we did not exclude any type of elder care service from participating in the study. Six elder care services, all part of the municipal elder care system, agreed to participate: Two RCHs, two assisted living facilities (ALF), and two home help services. Swedish RCHs are residential long-term facilities where residents live in individual apartments, with round-the-clock access to care staff assisting with, for example, administration of medication, hygiene routines, and preparation of food, and physicians are available on-call (Nilsen et al., 2018). ALFs are also residential long-term facilities but cater to residents with fewer medical needs than in RCHs, providing supported accommodation with care staff available. Home help, or home care services, aids with daily chores such as cooking, washing, and cleaning in older peoples’ own homes. In the second recruitment phase, contact persons at each service invited individual staff members to the workshop series. The workshops were open to all elder care staff, regardless of profession, and the only inclusion criterion was having at least 6 months work experience. Invitations were either made face-to-face or via email. Five workshop series were conducted with the six participating services. As shown in Table 1, the first workshop group was comprised of staff from two services while subsequent groups consisted of staff from one service in each.

**Table 1.** Overview of Groups Participating in the Workshop Series.

Group	Service	Participants	Median attendance (range)
1	RCH A, home help A	4	3 (1–4)
2	ALF A	9	7 (5–8)
3	ALF B	9	8 (7–9)
4	Home help B	6	5 (4–6)
5	RCH B	10	9 (9–10)

*Note.* ALF = assisted living facility; RCH = residential care home.

Written and oral information, including study purpose and methods, the right to withdraw at any time without consequences, and a request to audio-record workshops were received by all participants, and written informed consent was obtained for each workshop. While the risk of harm to participants was considered low, the facilitators were experienced in dealing with emotional reactions to the topics and had the means to refer participants to third-party support if needed. One participant chose to withdraw during the third workshop due to personal circumstances they described as making continued participation too emotional. During a follow-up conversation, this participant clarified appreciation of the workshops, saying that further support was unnecessary. Another participant withdrew following the first workshop, as the participant had expected the series to involve formal training in palliative care. Two invited participants did not attend any of the workshops, with no reasons given.

### Workshop Procedure and Data Collection

**Figure 1.**
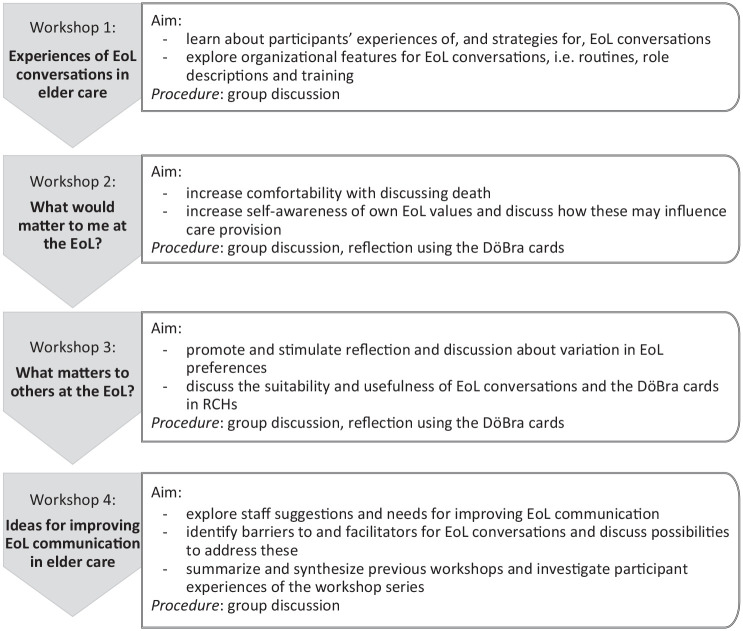
Overview of the workshop series and individual workshop aims. *Note.* EoL = end-of-life; RCH = residential care home.

As illustrated in Figure 1, we conducted a series of four consecutive workshops with elder care staff to promote EoL communication in RCHs by: exploring prerequisites for EoL conversations; discussing one’s own and others’ EoL values; and supporting preparedness for engaging in EoL conversations with residents and their families. The workshops were designed to integrate individual and joint reflective exercises with group discussions to stimulate reflection and knowledge exchange in the groups. Each workshop in the series was guided by a theme.

The first three workshops in each series were held approximately 2 weeks apart, with the fourth, final workshop 3–4 weeks after the third. The workshops were held between May 2017 and March 2018 in conference rooms at the services. Each workshop was 2 hr long with a break in the middle. The length of the workshops ranged from 73 to 115 min, with an average length of 99 min. All workshops were audio-recorded and professionally transcribed verbatim. Following PAR and adhering to the researcher flexibility encouraged in longitudinal qualitative studies (Saldaña, 2003), semistructured workshop guides were iteratively developed throughout the study to incorporate new perspectives and issues and adapt the workshop format to be as relevant as possible. Each individual workshop and each series thus informed the next.

The workshops were facilitated by two women: Authors Ida Goliath, PhD and registered nurse with a background in EoL care research and practice and extensive experience as a facilitator and interviewer, and Therese Johansson, a doctoral student with an MSc in psychology, also an experienced interviewer. Both were unknown to the participants and had no other relationship with the involved services. Workshops began by discussing expectations and/or reflections from the last workshop, before introducing the theme. Discussions were facilitated with two main strategies: “round-the-table discussions” allowing all participants to speak and “popcorn style”, using open questions to freely trigger reflection, sharing, and discussions. Each workshop ended with a closing round in which each participant was asked to share their reflections about the session, in line with recommendations for longitudinal qualitative studies in care services (Calman et al., 2013). See Supplement file 1 for an example of the workshop procedure.

To stimulate reflection among participants, Workshops 2 and 3 incorporated reflective exercises using the DöBra cards, a translated and adapted Swedish version of the U.S. English-language GoWish cards (Menkin, 2007), designed to support and structure EoL conversations (Lankarani-Fard et al., 2010). The DöBra deck consists of 37 cards, each with a statement that can be considered important at the EoL (Steinhauser et al., 2000). Statements cover a wide variety of potential preferences related to physical, practical, existential, and social matters at the EoL (see Tishelman et al., 2019 for card statements). There are also three “wild cards” that can be used to include any priorities not covered in the preformulated cards. The GoWish cards and the DöBra cards follow the same procedure. Individual cards are first sorted into three piles based on their perceived importance. The cards deemed most important are then ranked according to their priority, from 1 to 10. The original GoWish cards have been used in both clinical and educational contexts (Lankarani-Fard et al., 2010; Menkin, 2007; Osman et al., 2018) and the Swedish DöBra cards have been found to be an easy-to-use and accepted tool for stimulating reflection among community-dwelling older adults without known palliative care needs (Eneslätt et al., 2020; Tishelman et al., 2019).

In Workshop 2, participants used the DöBra cards individually to reflect on what would matter to them personally. In Workshop 3, they were used individually or in Groups of 2 and 3 to reflect on a specific resident’s preferences. In both workshops, participants followed the instructions outlined above. Participants’ reasoning underlying prioritizations were then discussed together to further explore and reflect on EoL values and preferences.

### Data Analysis

We were inspired by Saldaña’s (2003) approach to longitudinal qualitative analysis, focusing on identifying and analyzing changes in data over time. Through data analysis, we came to conceptualize change as a gradual and dynamic process, demonstrated by participant reflections and interactions throughout the workshop series. Each group of participants was considered a case and each workshop an observation. Analysis of change over time was guided by asking framing questions, that is, “what is different between one observation to the next?” and “what increases or decreases over time?” (Saldaña, 2003).

Inductive analysis was initiated by repeatedly listening to and reading the 20 workshop transcripts to become familiar with the data. The first cycle of coding involved inductive indexing and sorting of data based on content in NVivo. Pro (version 11). Content-based codes, for example, *routines for EoL care*, were combined into clusters based on commonalities, which were in turn organized into eight broader categories, for example, *death as part of the job*. This initial coding structure focused primarily on factors that facilitated or hindered EoL communication in elder care. Group interactions and contextual conditions were also considered in this process.

Reviewing this first coding structure initiated the second cycle of coding, by reanalyzing data and revising the initial coding structure with an increased focus on identifying changes in how EoL values and communication were discussed in the groups. A data matrix was constructed to distinguish changes both within and between cases over time. This representation of data added a chronological examination to reveal links or interrelations between observations (Saldaña, 2003). This second cycle allowed reworking and collating categories into five manifest themes (Saldaña, 2013), which covered processes of change.

**Figure 2.**
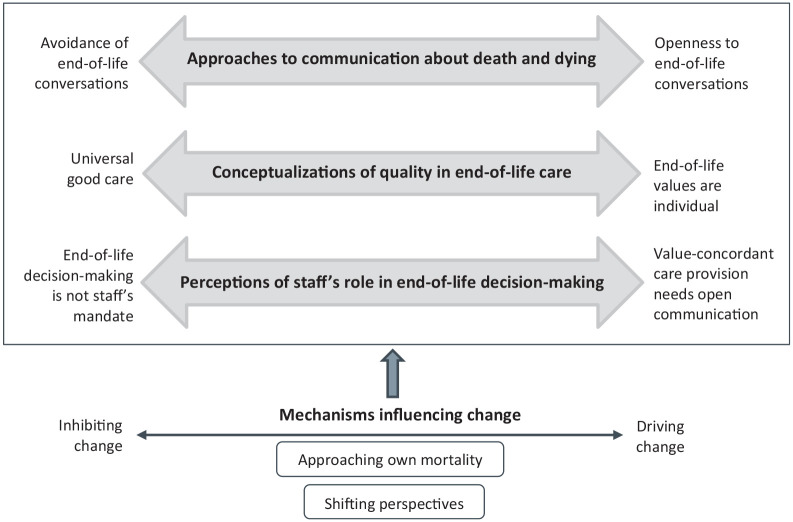
Illustration of change continuums within three themes and the underlying mechanisms influencing change.

In a third cycle, themes were revised and refined by all authors through repeated discussions to incorporate commonalities and differences in changes in and across cases, becoming more interpretive. The coding scheme consisted of three themes and is demonstrated in Supplement file 2. To illustrate the breadth of change observed in the data, themes were conceptualized as continuums of change, as illustrated in Figure 2.

There is always a risk that researchers’ preconceptions and biases influence data interpretation (Morse, 2015). We, therefore, made various efforts to uphold analytic rigor. Following each workshop, the facilitators held reflexivity briefings to summarize the workshop discussions, reflect on facilitation, and consider if and how future workshops should be adapted. Throughout the analytic process, reflections and preliminary ideas about data were documented, creating an extensive audit trail. As both authors who acted as workshop facilitators led the analysis, they knew the data well and could discuss alternative interpretations. Preliminary findings were critically reviewed in the multidisciplinary author group to guard against selective use of data and unsubstantiated interpretative claims (Calman et al., 2013). Analytic points were supported by numerous quotes from the data, with illustrative examples chosen for presentation.

## Results

In total, 38 staff members participated in the workshops. Attendance varied over the course of the workshop series, for example, due to daily staffing levels and sick leave, with 1–10 participants (median=6) in each workshop. All workshop groups combined professions, except Group 5, which consisted of ANs only. Few participants knew each other prior to the workshops as they generally worked in different units of the service. Although participants were primarily women and ANs, they were diverse in age (range 23–65 years), work experience (range 0.5–41 years), and place of birth (15 countries, including Sweden). Participant demographics are presented in full in Supplement file 3.

We identified three themes illustrating continuums of change in staff discussions over time (Figure 2). These themes are not mutually exclusive but presented separately here for clarity. The themes were all influenced by underlying mechanisms that influenced change, either by driving or inhibiting change processes. Changes were not necessarily demonstrated by all participants or in all groups but constitute overall patterns in the data. We begin each theme with a short summary, substantiated thereafter in more detail with examples from the data and followed by a discussion of findings related to underlying mechanisms affecting change processes. Sources of quotes are noted by group number and workshop number in the series of four.

### Theme 1: Changes in Approaches to Communication About Death and Dying

The first theme describes changes over time in participants’ reflections and discussions about EoL conversations in the elder care context, along a continuum from avoidance to openness about engaging in conversations. In many, but not all, groups, the process of reflecting on and discussing one’s own EoL values and experiences served as learning opportunities that increased awareness of openings to initiate EoL conversations with residents or relatives and preparedness for engaging in them. In addition, participants noted feeling more at ease and confident when death was brought up in daily work.

Overall, the phenomenon of death described as both a natural part of work and a topic rarely addressed, as noted in the literature above (Almack et al., 2012; Österlind et al., 2011), was prominent here. The EoL was depicted as charged with negative associations and emotions, making it difficult to broach. In the first workshops of the series, participants often argued that raising EoL issues with older, frail residents nearing the EoL was not suitable as it could cause distress. In some facilities, such opinions were said to be ingrained in the work culture:

*I feel like. . .that there is a climate* [in the facility] *that you don’t want* [. . .] *residents to become worried or sad* [. . .] *my experience is that you should avoid* [mentioning death]. *You sweep it under the carpet.* (Group 3 (G3), Workshop 1 (W1))

Few participants described experiences of open communication about EoL matters with residents and/or their families. However, residents’ commenting about death was said to be common, though this was generally interpreted as a symptom of depression or an attention-seeking tactic. Residents were thus not considered to genuinely wish to talk about the EoL and participants described how, rather than continue the conversation, they would normally respond with different strategies to distract or comfort the resident to assure their wellbeing. At the same time, participants also suggested that residents are likely to be aware that they will die soon and may have thoughts about the EoL that they would like to discuss but do not raise:

*It’s probably completely obvious to most people who move here, that this is my last move. You’re in that stage of life* [. . .]. [The residents] *are aware of this too, but I have a feeling that. . .they don’t want to bother us so much with this.* (G2, W1)

Nonetheless, there were concerns that if staff were to initiate EoL conversations, they may: “*introduce thoughts to* [residents], *that they maybe don’t have*” (G3, W1). This form of mutual protection between residents and staff appeared to contribute to the avoidance of EoL issues altogether.

However, the idea that mentioning death was intrinsically harmful, became increasingly challenged throughout the workshop series. During the reflective exercises in Workshop 2, several participants stated that they would appreciate the opportunity to discuss their own EoL preferences, to prepare themselves and those close to them: “*I’d probably be happy if someone asked me* [about my EoL preferences] [. . .] *it’s essentially about some kind of comfort, both for the person who is dying and for those who are left*” (G4, W2)

Stimulated by Workshop 3’s reflective exercises about residents’ EoL preferences, experiences of situations in which residents mentioned death and dying were revisited in several groups. Other aspects were now raised by participants, for example, that residents may find it comforting to share their thoughts about death. Some participants reflected on their own behaviors, for example, how they might have previously missed residents’ invitations to EoL conversations or deliberately avoided or discouraged them. Increasingly, there was debate as to whether protecting residents from EoL discussions might even be harmful as it risked leaving residents feeling unseen or unheard, without a chance to vent their thoughts: “*it can be very healthy* [. . .] *to feel worried and sad and to let that out*” (G2, W4)

In the later workshops in the series, participants tended to demonstrate a sense of increased comfort, preparedness, and openness to engage in EoL conversations:

*I felt relieved actually, something that is very frightening at first, when you go into* [the workshop series] *then all of a sudden you get another perspective. It’s not as frightening as you felt it was before. Like when you walk into a dark room, in the beginning you can’t see, and it’s scary. But then you get used to it, and it feels a little better. You get a little sense of security. . .* (G5, W4)

Although some participants still maintained that the EoL was a difficult topic, the workshops encouraged and empowered participants to personally reflect on death, by sharing personal, sometimes difficult experiences. This sharing of experiences appeared to make participants feel more at ease and EoL conversations less negatively charged. Group 3 was an outlier in that they demonstrated little change, with several participants remaining doubtful that staff themselves should address EoL issues, suggesting instead that other, more specialized professionals, for example therapists or clergy, were more suitable.

During the course of the workshop series, the reflective exercises were described as insightful and triggering new curiosity about the EoL values of others. Several participants expressed desires to have EoL conversations at home or with friends, with some borrowing the DöBra cards. However, participants said they could initially be met with reluctance when raising issues of death and dying in their private lives:

*I tried to talk with my husband, who doesn’t want to talk about death* [. . .] [I thought] *I shouldn’t push him, but two days later he came to me himself, asking “what are these cards about?”* [. . .] *we’ve been married for 13 years and we’ve never talked about death.* (G5, W4)

Relating this incident to the group highlighted how an initial dismissal of an EoL conversation was susceptible to change, not necessarily indicating a lack of interest.

### Theme 2: Changes in Conceptualizations of Quality in EoL Care

The second theme covers changes throughout the workshop series in participants’ conceptualizations of what good EoL care entails, from initial discussion of EoL values as universal toward increasing recognition that quality relates to individual values and preferences. Workshop 2’s reflective exercises about one’s own values appeared to trigger discussion of both similarities and differences in what mattered to participants at the EoL and why, which then carried over to Workshop 3’s discussion of residents’ preferences.

While initially sharing their EoL care experiences, participants often expressed confidence in providing care when a resident showed signs of nearing the EoL. Discussions focused primarily on descriptions of bodily care, for example, preventing bed sores, or performing oral hygiene, and care after death, for example, dressing the dead body according to wishes. At this time, participants generally seemed to conceptualize quality EoL care as meeting a set of universal human needs and values, often related to “not dying alone” and “not dying in pain.”

Participants also discussed how care provision for an individual resident was guided by getting to know them over time, as one AN reflected:

*If you’ve* [cared for] *someone for a long time, and you get to* [the EoL] *stage, then you know this person a little* [. . .] *and maybe you know the relatives and what they think and how* [residents] *themselves want you to act.* (G2, W1)

However, staff members’ relationships with residents were generally focused on “doing things” for the resident and values seemed largely tacitly inferred from assumptions and interpretation of body language and facial expressions, rather than from explicit conversation.

In the reflective exercise in Workshop 2, participants were asked to present their own most prioritized DöBra card preferences to the rest of the group. In comparison to initial discussions about quality EoL care in the first workshop, card statements now triggered a range of stories about experiences. For example, the card “Not dying alone” catalyzed stories about residents dying when relatives briefly left the room, which led to further, more analytic discussion, for example, whether it might be difficult to die with others watching, and nuances between “being alone” and “feeling alone.” Through such discussions, participants increasingly included and emphasized individual values when discussing how EoL care should be informed.

Awareness of variation in EoL preferences was further demonstrated in Workshop 3, when participants used the DöBra cards to reflect on a specific resident’s EoL values. This exercise was generally described as more difficult than the self-reflection in Workshop 2 with participants emphasizing repeatedly that their decisions were based on assumptions and guesswork. An AN considers challenges in prioritizing preferences of a specific resident, saying:

*I know who he is, but I’ve only made small talk. I actually have no idea* [what’s important to him], *I’ve only seen the facade, the outside. It’s really difficult* [to know]. *And even if we were to choose* [a resident] *I know very well, I think it’d still be difficult anyway, because most people don’t broadcast their innermost* [values], *I don’t think. . .* (G5, W3)

This reflective exercise also served to make participants more aware of how values may be erroneously assumed. In the later workshops, it was recognized that present EoL care provision may be more informed by RCH routines, staff assumptions, and relatives’ views than by the explicit preferences of the dying resident. Several participants began to argue that the only way for staff to learn about residents’ preferences is to ask directly, while others maintained that some EoL values are universal:

*Of course, we’re different, and we want different things in life and such, but for as long as I’ve been here* [. . .]. *No one wanted to be alone* [at the moment of death], *as I’ve seen, and there’ve been quite a few (G5, W4)*

Some groups showed clear disagreement as to whether values can be inferred from professional experience or not. Such controversy epitomized the differences in resistance to, or support for, conducting EoL conversations. However, a number of participants pointed out that even if most EoL care values might be universal, there are still exceptions that may be missed if residents are not asked.

### Theme 3: Changes in Perceptions of Staff’s Role in EoL Decision-Making

The third theme concerns changes in how participants discussed the role of staff in the decision-making process for future EoL care provision. Throughout the workshop series, participants progressively emphasized challenges in providing EoL care in accordance with the resident’s values if open communication about death and dying was lacking.

In the initial workshops, participants often stated that residents’ wishes determined care provision. Yet, residents’ active involvement in the decision-making process was rarely mentioned. Instead, relatives were described as having much influence on EoL care decisions in particular. Few participants had experienced conversations to plan EoL care in advance. Acknowledging a resident’s deteriorating condition was seen as difficult for family members, who often focused on rehabilitation. This was said to hinder staff from raising EoL issues in advance. If the EoL was at all addressed with resident’s family, it was described as occurring late, when care decisions were urgent. In all workshop groups, participants shared experiences about EoL care choices hastily discussed with distressed relatives who were unsure of, or disagreed about, the resident’s wishes. Such situations were said to be morally and emotionally challenging and participants described how they sometimes tried to negotiate when they thought that wishes or care demands were not in the resident’s best interest. However, many said they struggled with the extent of their mandate in such interactions, and some described complying with relatives to avoid complaints or conflicts. This is illustrated here by a participant sharing an interaction with a relative who demanded that her frail mother should be physically activated:

*We* [staff] *knew that she can’t* [get out of bed]. *But what do we say to the relatives? You can say “okay, we will try to help her*,” *but even when she had just arrived, we knew* [she was dying]. . . (G5, W1)

Several participants suggested that although engaging in EoL conversations proactively could aid future decision-making by clarifying care preferences, addressing the EoL too soon could endanger trusting relationships with both residents and relatives, as exemplified in this interaction between two ANs about the timing of such conversations:

*AN1: . . .I’m thinking about the end, how they want it to be. Asking* [residents]*”do you want to talk about* [the EoL]*? Do you want us to talk to your family members?” earlier.*

*AN2: Are residents ready for that when they move here? It’s a huge step to arrive here.* [. . .] *it’s asking too much.* (G2, W3)

As the workshops progressed, participants expressed less apprehension about harming their relationships with residents or relatives. Instead, there was increasing emphasis on the value of involving them in EoL conversations to establish open communication and support preparation for the EoL. However, as exemplified above, one persistent concern, raised in all workshop groups, was when and how to address EoL care issues with a resident or relative without being confrontational. Participants talked about having inadequate experience and skills to support them in determining this balance in initiating discussions:

*I think it’s really important to have* [early EoL conversations] *actually* [. . .] *Because either way, you need to know* [about preferences]. *In some way it must come up, so that you don’t do things that are totally wrong* [. . .] *These are things that you otherwise don’t know. . . that relatives don’t talk about amongst themselves either.* (G5, W3)

However, some participants questioned if initiating EoL conversations should be the responsibility of staff, instead suggesting that this responsibility was in the hands of residents and their family members. Participants described themselves as lacking both time, training, and mandate, relating their professional behaviors more to their individual level of comfort and personal experiences of EoL situations within their own families.

### Mechanisms Influencing Change

Overall, we found two common underlying mechanisms that seemed to affect the thematic changes described above: “Approaching one’s own mortality” and “shifting perspectives,” which included reevaluating one’s own behavior and assumptions. These mechanisms, which became most clear during the reflective exercises with the DöBra cards in Workshops 2 and 3, seemed able to both drive and inhibit change processes.

There seemed both a cognitive and an emotional facet to approaching one’s own mortality. Participants noted that using the DöBra cards provided a vocabulary and structure, that is, cognitive tools, to reflect on EoL values more tangibly, allowing deeper introspection. The variation in card statements was described as enabling participants to approach their own mortality more easily, both cognitively and emotionally, bringing death closer in a way that was new to most. The emotional component was further illustrated as participants described the self-reflective exercise in Workshop 2 as a moving experience that brought up thoughts and feelings which they might otherwise suppress. While there were some participants who expressed not wanting to delve into their future EoL, often because it was emotionally charged, the experience was described by many as meaningful, insightful, and empowering: “*It feels really good in a way. A bit liberating”* (G5, W2). Another participant shared: “[It’s] *an eye opener, maybe I should write* [my priorities] *down.*” (G3, W2). Participants with a strong emotional response to the reflective exercises seemed to also identify discussions about death and dying as a salient issue in subsequent contact with residents and relatives. In contrast, in later workshops, it was most commonly those participants who had been uncomfortable talking about death themselves, who maintained that the EoL was not a suitable topic of discussion in elder care.

When reflecting on what they felt mattered to them or one of their residents, participants often described imagining EoL care from different viewpoints, for example, as a resident or a relative. Shifting perspectives in this way enabled participants to review and analyze their own and others’ behavior in EoL situations. By doing so, this mechanism also brought an opportunity for extrospection, for example, question previously taken-for-granted assumptions in relation to the EoL. For example, while speaking about their own EoL preferences in Workshop 2, some participants expressed concerns about maintaining their future autonomy and dignity if institutionalized. Such comments led to discussions about how colleagues would sometimes inadvertently disregard wishes, coax, or make decisions for residents in everyday care. Thus, by shifting perspectives, participants extended their frames of reference, often making staff’s own influence in care provision more visible. One AN summarized her own change process, saying: “*actually, now I think about how many* [staff members] *have avoided* [death] *all these years, and I’ve been one of them myself before, but I don’t do that anymore”* (G1, W4). Overall, these mechanisms appeared to play a role in driving changes by reinforcing care ideals and stimulating professional development, though mechanisms occasionally led to a focus on barriers for EoL conversations, which impeded further discussion.

## Discussion

Through longitudinal qualitative analysis of transcripts from a series of workshops using reflection and knowledge exchange to promote EoL communication in elder care, we identified three overarching continuums of change in participants’ reasoning over time. A first continuum concerns changes in how communication about dying and death came to be seen as more feasible, salient, and valuable. A second relates to shifts from a priori and generalized conceptualizations of what constitutes quality in EoL care, to conceptualizations based on consideration of individual variation in values. A third continuum concerns changes in awareness of staff’s roles and responsibilities in facilitating open communication with residents and their relatives as a prerequisite for EoL decision-making in accordance with each resident’s values. Two main underlying mechanisms were identified as driving or inhibiting these changes throughout the workshops: cognitive and/or emotional approaching of one’s own mortality; and shifting perspectives about EoL care, that is, imagining different viewpoints, questioning own assumptions, and analyzing one’s own behavior. In this study, the use of the DöBra cards as a tool in the workshops prompted reflection and served as a basis for discussion about EoL care and communication.

Our study contributes to a fuller understanding of features involved in developing staff competence for EoL conversations. The initial staff discussions in the workshops corroborated a recurrent problem noted in the international literature, that is, a silence surrounding death, which may be assimilated into, and reinforced by, the work culture (Alftberg et al., 2018; Omori et al., 2020). Throughout the workshop series, however, participants became more supportive of, and explicitly expressed feeling more prepared for, EoL conversations, although several stated they still did not feel entirely comfortable about initiating conversations themselves. Still, the workshops seemed to provide an opportunity for staff to reflect on the discourse of death in their respective workplaces, prompt self-awareness of the lack of communication, and initiate discussion about potential ways to address the EoL in a non-confrontative manner.

Competence in EoL care extends beyond factual and practical knowledge, encompassing also what has been described as “self-competence,” which relates to personal features, that is, values and fears that may influence behavior (Chan & Tin, 2012). However, much care staff education still implicitly relies on a so-called knowledge, attitudes, practice model, which posits that increased knowledge leads to changes in attitudes and subsequently changes in practice (Diwan et al., 1997). This linear didactic model has been increasingly criticized in favor of experiential pedagogical approaches based instead on participation, collaboration, and reflection (Diwan et al., 1997; Filmer & Herbig, 2020). In care contexts, educational approaches that rely on reflection and experiential learning are seen to support staff in examining and disentangling personal EoL views from their professional roles, reduce reluctance to discuss death, and develop competence in EoL communication (Doka, 2015; McClatchey & King, 2015). The mechanisms described in our study provide further detail and insight into the ways in which learning, and empowerment, can be fostered for this purpose.

There may be several, possibly interrelated, explanations for the observed changes in staff approaches to EoL conversations over the course of the workshop series, particularly in relation to increasing openness and reducing apprehension. Considering that many participants stated that death and dying were not something they talked about in the workplace, repeated participation in reflection and discussion about EoL communication at the service may have increased the emotional salience and professional relevance of EoL conversations per se (see Jones et al., 2020). The workshop format may also have served as a forum for staff to address their own emotions related to the EoL. Death anxiety due to exposure to sickness and death is not uncommon (Nia et al., 2016) and care staff can struggle to balance their professional and personal identity when providing EoL care (Broom et al., 2015; Funk et al., 2017). Indeed, in all groups, notable EoL experiences were spontaneously shared during the discussions. Participants’ initial apprehension about engaging in EoL conversations may also have been assuaged by the acquisition of a vocabulary for thinking and talking about dying and death, making it easier to share and ask each other about previous experiences, as well as identify issues and suggest improvements (Sand et al., 2018). The varying extent to which the mechanisms of change were demonstrated in the groups suggests that the reflective exercises may have affected participants differently. Reflection can be conceptualized as having a vertical dimension, with higher levels indicating deeper analysis and critical reflection that is conducive for learning (Moon, 2004). By integrating processes of introspection, involving attention to own feelings, attitudes, and values, and extrospection, focusing on reevaluating previous incidents and learning from others’ experiences, the mechanisms identified in our study may have triggered higher levels of reflection among some participants.

Death literacy may be seen as one goal of competence-building initiatives, such as the one described in our study. This concept, comparable to health literacy, has recently been defined as a set of experience-based knowledge and skills needed to access, understand and make informed choices about EoL care that strengthens caring capacity (Leonard et al., 2020).

The change continuums identified through analysis of data from the workshop series illustrate increasing acknowledgment that EoL conversations can be both valuable for informing and ensuring future value-concordant care, as well as cultivating and supporting EoL preparation for both residents, relatives, and staff. Our results add precision in identifying changes with the potential to support death literacy that are relevant to consider when designing death education initiatives.

Still, there are points of contention that were not resolved through staff reflection and knowledge exchange in our workshop series, particularly relating to a lack of clarity about optimal timing for EoL conversations and questions about responsibility and mandate for initiating them. The issue of timing has consistently been shown to hinder proactive EoL conversations in several care settings (Im et al., 2019; Niranjan et al., 2018; Rietjens et al., 2021). The perceived lack of clarity about mandate links to an area that permeated all themes, related to increasing awareness and discussion of the need for organizational support for conducting EoL conversations systematically. Contextual conditions, that is, resources, time, and work culture, are known to influence the impact and sustainability of competence-building initiatives in elder care (Frey et al., 2019; Gilissen et al., 2017; Nilsen et al., 2018), appearing able to both inhibit and facilitate change processes in practice.

### Limitations

Our study also has a number of limitations. Although the workshop groups comprised different types of services and demographically varied staff, all were part of the same municipal care system, and thus, share some aspects of organizational culture.

While efforts were made to illustrate our points with data from a variety of participants in all workshop groups, Group 5 is quoted more often here, in part due to its consistently high attendance, lively discussions, and participants who expressed themselves more succinctly in Swedish. Nevertheless, the quotes in the text represent patterns in the data set as a whole as workshop discussions featured similar descriptions of EoL communication experiences and perceptions of obstacles and prerequisites for EoL conversations across the groups, despite the different contexts. It did become clear, however, that the time restriction for home help visits meant that staff had little possibility to engage in EoL conversations, had they wanted to.

The iterative nature of PAR means that researchers are directly involved in and influence the research process (Baum et al., 2006); latter groups may, therefore, have benefited from the facilitators’ experience in adapting the workshops to be more relevant. It is also important to note that our study does not allow conclusions on impact in actual practice to be drawn, that is, whether changes led to an actual increase in the incidence of EoL conversations or had other long-term effects on EoL communication. The collective learning and empowerment observed in the workshop series may still influence practice in less tangible ways though, for example, by initiating change in the shared social reasoning about the EoL in services (Stuttaford & Coe, 2007).

## Conclusion

Competence-building is a multifaceted and relational concept that encompasses knowledge, skills, and empowerment among staff, and requires support from the organization. Our study suggests that an approach to staff competence-building for EoL conversations based on repeated reflection, discussions, and knowledge exchange, can support changes in: staff approaches to EoL communication; assumptions about what constitutes quality in EoL care; and acknowledgment of staffs’ own roles in EoL decision-making processes. Individual and joint reflection, using an appropriate and user-friendly tool, enabled staff to approach their own mortality and expand their frames of reference by shifting perspectives of EoL care, which were important mechanisms of change in this study.

Our results add relevant nuance and detail about how reflection, involving introspection and extrospection, can prompt experiential learning and may contribute to the development of death literacy. The change continuums presented here indicate core aspects to include in EoL competence-building programs and death education, whereas the mechanisms provide insight into how death literacy might be fostered. These findings are important to consider in future educational initiatives to improve EoL communication between stakeholders in various care contexts. Nevertheless, the question of whether increased death literacy translates to changes in staff behavior in care practice remains critical for future research to explore.

## Supplemental Material

sj-pdf-1-qhr-10.1177_10497323211012986 – Supplemental material for Continuums of Change in a Competence-Building Initiative Addressing End-of-Life Communication in Swedish Elder Care Click here for additional data file. Supplemental material, sj-pdf-1-qhr-10.1177_10497323211012986 for Continuums of Change in a Competence-Building Initiative Addressing End-of-Life Communication in Swedish Elder Care by Therese Johansson, Carol Tishelman, Joachim Cohen, Lars E. Eriksson and Ida Goliath in Qualitative Health Research

sj-pdf-2-qhr-10.1177_10497323211012986 – Supplemental material for Continuums of Change in a Competence-Building Initiative Addressing End-of-Life Communication in Swedish Elder Care Click here for additional data file. Supplemental material, sj-pdf-2-qhr-10.1177_10497323211012986 for Continuums of Change in a Competence-Building Initiative Addressing End-of-Life Communication in Swedish Elder Care by Therese Johansson, Carol Tishelman, Joachim Cohen, Lars E. Eriksson and Ida Goliath in Qualitative Health Research

sj-pdf-3-qhr-10.1177_10497323211012986 – Supplemental material for Continuums of Change in a Competence-Building Initiative Addressing End-of-Life Communication in Swedish Elder Care Click here for additional data file. Supplemental material, sj-pdf-3-qhr-10.1177_10497323211012986 for Continuums of Change in a Competence-Building Initiative Addressing End-of-Life Communication in Swedish Elder Care by Therese Johansson, Carol Tishelman, Joachim Cohen, Lars E. Eriksson and Ida Goliath in Qualitative Health Research

## Footnotes

* DöBra is a Swedish play on words, literally meaning “dying well” but figuratively meaning “awesome”.
