# Supplementary material for: Continuums of Change in a Competence-Building Initiative Addressing End-of-Life Communication in Swedish Elder Care
Source: Qual Health Res. 2021 May 13;31(10):1904–17. doi: 10.1177/10497323211012986 (PMC8446900; doi:10.1177/10497323211012986)
Supplement: sj-pdf-1-qhr-10.1177_10497323211012986 – Supplemental material for Continuums of Change in a Competence-Building Initiative Addressing End-of-Life Communication in Swedish Elder Care [file sj-pdf-1-qhr-10.1177_10497323211012986.pdf]

Supplement file 1. Example guide for workshop facilitators based on Workshop 2, Group 5

General information about the workshop series

- Ground rules for the discussion
  - There are no right or wrong answers – we may have different opinions
  - Let others finish what they are saying before you speak
  - It is ok to ask each other questions
  - We are mindful about not sharing personal stories outside the workshop

(The outline below indicates general topics to be covered in the workshop. Follow-up questions posed by the facilitators include, e.g.: *In what way? Can you tell more/expand and/or give examples? What do you mean? What are your reflections about that? Does anyone have questions in relation to what xx just told us/talked about?* Questions may be repeated to allow several group participants to respond, if a discussion does not occur spontaneously.)

Introduction

- Participants' expectations of the workshop
- Reflections or questions since last workshop

Working with people nearing the end of life (EoL)

- What do you consider important when caring for people at the EoL?
- Can you tell us about a positive experience you have had of EoL care?
- Is there anything you would want to change about EoL care in your workplace?

EoL communication

- In what ways do residents mention dying or death? How do you respond?
- Do relatives address the EoL? How do you respond?
- How comfortable are you with talking about matters related to dying and death?
- What are your experiences of residents communicating wishes for their EoL?
- How are care preferences documented? Who has access to this information?
- How are disagreements related to treatment at the EoL handled?

Using the DöBra cards to reflect about own EOL values

Facilitators go through instructions on how to use the DöBra cards. Participants then use them individually.

- Round-the-table presentation of which three card statements were prioritized as the most important, and, if participants want to share, why these were chosen.
- Were there any statements that you found important or surprising?
- What are your reflections on the values and preferences of your colleagues in the group?
- What are your reflections about the DöBra cards? How did you experience using them?

Concluding reflection (round-the-table)

- What are your reflections about today's workshop?

Participants are reminded of the timepoint for the next workshop and encouraged to contact the facilitators if they have any questions, comments or reflections.
