# Supplementary material for: Continuums of Change in a Competence-Building Initiative Addressing End-of-Life Communication in Swedish Elder Care
Source: Qual Health Res. 2021 May 13;31(10):1904–17. doi: 10.1177/10497323211012986 (PMC8446900; doi:10.1177/10497323211012986)
Supplement: sj-pdf-2-qhr-10.1177_10497323211012986 – Supplemental material for Continuums of Change in a Competence-Building Initiative Addressing End-of-Life Communication in Swedish Elder Care [file sj-pdf-2-qhr-10.1177_10497323211012986.pdf]

Supplement file 2. Examples of progression in analysis from data, to codes, categories, and themes.

Examples of data quoted in article text	***Descriptive codes***	***Categories***	***Theme***
"*I feel like…that there is a climate* [in the facility] *that you don't want* […] *residents to become worried or sad* […] *my experience is that you should avoid* [talking about death]. *You sweep it under the carpet.*" (Participant 7 (P7), group 3 (G3), Workshop 1 (W1))	• Death is upsetting<br>• Residents are vulnerable and need protection<br>• Work culture influences individual behavior	Avoiding addressing the EoL with residents	**Approaches to communication about death and dying**
"*It's probably completely obvious to most people who move here, that this is my last move. You're in that stage of life* […]. [The residents] *are aware of this too, but I have a feeling that.*" (P5, G2, W1)	• RCH as a site for dying<br>• Residents know that they are approaching the EoL	A general awareness about the EoL	
"*Of course, we're different, and we want different things in life and such, but for as long as I've been here* […]. *No one wanted to be alone* [at the moment of death], *as I've seen, and there've been quite a few*" (P9, G5, W4)	• Some values and preferences are seen as universal<br>• Relying on professional experience of EoL care	Inferring what matters to others	**Conceptualizations of quality in EoL care**
"*I know who he is, but I've only made small talk. I actually have no idea* [what's important to him], *I've only seen the facade, the outside. It's really difficult* [to know]. *And even if we were to choose* [a resident] *I know very well, I think it'd still be difficult anyway, because most people don't broadcast their innermost* [values], *I don't think…*" (P3, G5, W3)	• Residents may not volunteer their preferences<br>• Knowing a person does not necessarily mean knowing their individual values	You cannot know unless you ask	
"*We* [staff] *knew that she can't* [get out of bed]. *But what do we say to the relatives? You can say 'okay, we will try to help her', but even when she had just arrived, we knew* [she was dying]…" (P4, G5, W1)	• Relatives advocate for more active care than staff<br>• Staff try to comply to relatives' expectations for care	Relatives as primary decision-makers	**Perception of staff's role in EoL decision-making**
"*I think it's really important to have* [early EoL conversations] *actually* […] *Because either way, you need to know* [about preferences]. *In some way it must come up, so that you don't do things that are totally wrong* […] *These are things that you otherwise don't know*" (P5, G5, W3)	• Staff perceive a need to know residents' values and preferences<br>• Knowing about preferences prevents unwanted care	Promoting EoL conversations to aid decision-making	
