# Supplementary material for: Continuums of Change in a Competence-Building Initiative Addressing End-of-Life Communication in Swedish Elder Care
Source: Qual Health Res. 2021 May 13;31(10):1904–17. doi: 10.1177/10497323211012986 (PMC8446900; doi:10.1177/10497323211012986)
Supplement: sj-pdf-3-qhr-10.1177_10497323211012986 – Supplemental material for Continuums of Change in a Competence-Building Initiative Addressing End-of-Life Communication in Swedish Elder Care [file sj-pdf-3-qhr-10.1177_10497323211012986.pdf]

Supplement file 3. Demographic characteristics of participants in the workshop series.

Participants	*N=38*
**Gender**	
Women	32
Men	6
**Profession**	
Assistant nurse (AN)	26
Registered nurse (RN)	3
Nursing aide	2
Activity coordinator	3
Other	4
**Education (highest qualification)**	
Primary (9 yrs)	1
Upper secondary education	19
Higher vocational education diploma	4
University qualification (<3 yrs)	5
University diploma (>3 yrs)	9
**Place of birth**	
Sweden	19
Europe excl. Sweden	4
Africa	5
Asia	6
North America	2
South America	2
